# Comparison of nuclear texture analysis and image cytometric DNA analysis for the assessment of dysplasia in Barrett's oesophagus

**DOI:** 10.1038/bjc.2011.353

**Published:** 2011-09-20

**Authors:** J M Dunn, T Hveem, M Pretorius, D Oukrif, B Nielsen, F Albregtsen, L B Lovat, M R Novelli, H E Danielsen

**Affiliations:** 1 Department of Surgery, National Medical Laser Centre, University College London, London, UK; 2 Centre for Cancer Biomedicine, University of Oslo, Oslo, Norway; 3 Department of Histopathology, University College London, London, UK; 4 Institute for Medical Informatics, The Norwegian Radium Hospital, Oslo University Hospital, Oslo, Norway; 5 Department of Informatics, University of Oslo, Oslo, Norway

**Keywords:** Barrett's oesophagus, dysplasia, aneuploidy, image cytometry, nucleotyping

## Abstract

**Background:**

Dysplasia is a marker of cancer risk in Barrett's oesophagus (BO), but this risk is variable and diagnosis is subject to inter-observer variability. Cancer risk in BO is increased when chromosomal instability is present. Nucleotyping (NT) is a new method that uses high-resolution digital images of nuclei to assess chromatin organisation both quantitatively and qualitatively. We aimed to evaluate NT as a marker of dysplasia in BO and compare with image cytometric DNA analysis (ICM).

**Methods:**

In all, 120 patients with BO were studied. The non-dysplastic group (*n*=60) had specialised intestinal metaplasia only on two consecutive endoscopies after 51 months median follow-up (IQR=25–120 months). The dysplastic group (*n*=60) had high-grade dysplasia or carcinoma *in situ*. The two groups were then randomly assigned to a training set and a blinded test set in a 1 : 1 ratio. Image cytometric DNA analysis and NT was then carried out on Feulgen-stained nuclear monolayers.

**Results:**

The best-fit model for NT gave a correct classification rate (CCR) for the training set of 83%. The test set was then analysed using the same textural features and yielded a CCR of 78%. Image cytometric DNA analysis alone yielded a CCR of 73%. The combination of ICM and NT yielded a CCR of 84%.

**Conclusion:**

Nucleotyping differentiates dysplastic and non-dysplastic BO, with a greater sensitivity than ICM. A combination score based on both techniques performed better than either test in isolation. These data demonstrate that NT/ICM on nuclear monolayers is a very promising single platform test of genomic instability, which may aid pathologists in the diagnosis of dysplasia and has potential as a biomarker in BO.

The incidence of oesophageal adenocarcinoma is rising rapidly in the developed world. Barrett's oesophagus (BO) is a precursor lesion that confers a 30- to 100-fold increased risk of oesophageal adenocarcinoma above that for the general population, with incidence rates of 0.4–2% per annum in non-dysplastic BO (Cameron *et al*, 1985; Hameeteman *et al*, 1989; Thomas *et al*, 2007; Yousef *et al*, 2008; Sikkema *et al*, 2010). Progression appears to occur through a metaplasia–dysplasia–carcinoma sequence (Weston *et al*, 1999; Montgomery *et al*, 2001). High-grade dysplasia (HGD) confers a high probability of cancer, but rates of progression vary substantially in different studies with reported 5-year cumulative incidences of oesophageal adenocarcinoma ranging from <10–59% (Reid *et al*, 2000a; Buttar *et al*, 2001; Schnell *et al*, 2001; Overholt *et al*, 2007). The diagnosis of HGD is associated with inter-observer variation among community pathologists (Alikhan *et al*, 1999) as well as between specialist GI pathologists (Downs-Kelly *et al*, 2008).

There is much interest in the utility of molecular biomarkers in BO, both to predict which patients may develop cancer (and therefore offer therapy) and to aid prognostication by guiding surveillance intervals following therapy. Genomic instability seems to be a fundamental property of neoplastic progression that develops before the onset of cancer, and a large body of evidence now suggests that most oesophageal adenocarcinomas arise in association with a process of gain or loss of whole chromosomes or large portions of chromosomes (Reid *et al*, 2010). Abnormalities in DNA ploidy are a consequence of genomic instability that has been shown to predict future cancer risk in non-dysplastic BO when measured by flow cytometry, with a relative risk of 5.0 for aneuploidy (Reid *et al*, 2000b). Our group has also demonstrated that DNA ploidy, measured by image cytometric DNA analysis (ICM), predicts cancer progression in non-dysplastic BO following PDT, with a hazard ratio of 8.2 (Dunn *et al*, 2010). Image cytometric DNA analysis using digital images of Feulgen-stained nuclei is an accurate method to estimate DNA content, and comparable with flow cytometry on thick sections (Baldetorp *et al*, 1992; Kaern *et al*, 1992; Chen *et al*, 1995; Dunn *et al*, 2010). Advantages of ICM over flow cytometry include low set up cost, smaller number of nuclei required and greater sensitivity for non-diploid cell populations.

Nucleotyping (NT) is a methodology that uses powerful computers to interrogate nuclear DNA structure and organisation both quantitatively and qualitatively. Nucleotyping can be performed on the same high-resolution digital images of stained nuclei used for DNA ploidy analysis. As large-scale genomic instability correlates with large-scale re-arrangement of interphase nuclear chromatin, NT has potential as a single platform biomarker of cancer risk. Nuclear textural features have been shown to aid prognostication in several cancers including prostate, breast, head and neck and gynaecological tumours (Nielsen *et al*, 2008). The utility of NT for the assessment of BO has not been evaluated.

## Aims

The aim of this study was to evaluate NT for the diagnosis of dysplasia arising in BO and compare with DNA ploidy as measured by image cytometry.

## Patients and methods

### Patient selection

A total of 120 patients from the UCLH Barrett's Oesophagus cohort were included in the study. These were separated into two groups according to histology. The first group had specialised intestinal metaplasia (SIM) on four-quadrant biopsy at baseline surveillance endoscopy, which was confirmed by two specialist GI pathologists. To confirm that these patients were true non-dysplastic with low risk of progression, all had at least one follow-up surveillance endoscopy with a minimum follow-up of 2 years, which showed SIM only. The median follow-up was 51 months (IQR=25–120). The second group had HGD confirmed by two specialist GI pathologists. All patients were naive to endoscopic treatment at baseline. Analysis was undertaken on representative biopsies displaying SIM or HGD from one level of BO per patient. Two 40 *μ*m sections were cut from formalin-fixed paraffin-embedded (FFPE) tissue and then nuclear monolayers were prepared and stained with Feulgen as previously described (Pretorius *et al*, 2009).

### Digital image analysis

All the slides were studied using Nucleotyping Analysis System (Room 4, East Sussex, UK). This is an automated image cytometric analyser that consists of a microscope (Axioplan 2, Zeiss, Jena, Germany), a 546-nm green filter, and a black-and-white, high-resolution digital camera (AxioCam MRm, Zeiss). The pixel resolution obtained with this lens is 254 nm per pixel on the cell specimen. Fifteen hundred nuclei were automatically captured, measured and classified in each case. To exclude possible artefacts and non-representative nuclei (like doublets, necrotic or cut cells), all images were certified by trained personnel and stored in digital galleries.

### DNA ploidy analysis

Optical density and nuclear area were measured and integrated optical density of each nucleus was calculated as previously described (Kristensen *et al*, 2003). A histogram representing the DNA content was produced and analysed according to the European Society for Analytical Cellular Pathology guidelines (Haroske *et al*, 2001). Ploidy-related parameters such as DNA index and percentages of cells exceeding 5c (5c ER) and 9c (9c ER) were also noted.

### NT by grey level entropy matrices

**Figure 1:**
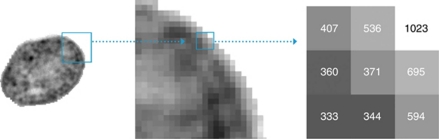
Example of a nucleus stained with Feulgen and captured by digital imaging. The schematic on the left demonstrates a window comprising nine pixels in a 3 × 3 square. The numbers correspond to the grey level of each individual pixel.

When analysing digital images of nuclear monolayers stained with Feulgen, the measured intensity of light is proportional to the DNA content at each pixel position and is referred to as the grey level. The higher-order statistical analysis is performed on square-shaped groups of pixels (called windows, see Figure 1). Window size defines the size of elements in the cell nuclei being described.

In order to characterise the distribution of grey levels within all such windows of an image, the grey level entropy matrix (GLEM) was defined (Yogesan *et al*, 1996). The matrix element *P*(*i, j∣w*) contains the estimated probability of a first-order grey level entropy value *j* within a window of size *w* × *w* centred around a pixel with grey level value *i* (Yogesan *et al*, 1996). Entropy is a measure of uniformity, so homogeneous image structures will give low entropy values whereas inhomogeneous structures will give high entropy values. Thus, the GLEM will describe both the distribution of local entropies and the distribution of grey levels in a given image. This is a way to quantify differences in chromatin structure throughout the nucleus.

The logarithmic entropy used here is defined as



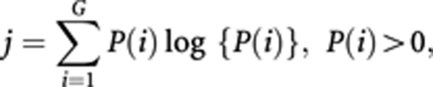



where *P*(*i*) is the normalised frequency of occurrence of grey level *i* within a window of size *w* × *w* and *G* is the number of grey level quantisation levels in the image. In this study, the number of grey levels in the image was reduced by re-quantisation from 1024 to 64 before the computation of the matrices, and the matrices were computed with a window size of 9 × 9 pixels.

### Adaptive textural features

In a previous study (Yogesan *et al*, 1996), nine features based on the GLEM were defined, and were subsequently shown to be of prognostic value in prostate cancer (Jorgensen *et al*, 1996). These nine features are weighted sums of the normalised GLEM element values, based on relatively simple weight functions. In a unified approach to statistical texture feature extraction, we have proposed a new method to extract only two adaptive features (Albregtsen and Nielsen, 2000; Albregtsen *et al*, 2000; Nielsen *et al*, 2001). By using an adaptive weight function, extra weight is given to matrix elements that discriminate well between the two classes. For each patient, the nuclear images were grouped into area intervals according to the number of pixels in the image (*A*_0_=<1000 pixels, *A*_1_=1000–1999 pixels, *A*_2_=2000–2999 pixels, … , *A*_10_=>10 000 pixels) (Nielsen and Danielsen, 2006). Within each of these area groups (*A*_*a*_), the average entropy matrix *P*(*i*, *j∣w, A*_*a*_*, ω*_*c*_) and the variance entropy matrix *σ*^2^(*i*, *j*∣*w*, *A*_*a*_, *ω*_*c*_) were computed for each of the two classes, *ω*_1_ and *ω*_2_. We then calculated average matrices 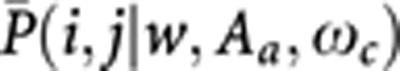 and 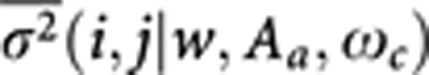 over all the learning set cases in each class *ω*_c_, for each of the area intervals. Using this two-step procedure, we avoid biasing the average and variance by an eventual uneven number of nuclei in each class and area interval. Based on these matrices, we computed a class difference matrix and a squared Mahalanobis class distance matrix, that is, the squared difference between the two class element values, divided by the average of the two class variances for that particular GLEM element (Nielsen and Danielsen, 2006). Using these class difference and distance matrices, we extracted two adaptive features from each nucleus, using the squared elemental Mahalanobis distance as a weight in a weighted summation of the GLEM, summed over the positive/negative partition of the class difference matrix, respectively. Finally, mean feature values were calculated for each patient from all nuclei defined by the area intervals *A*_*a*_, where *a*=1, 2, … , 5. The result is a low-dimensional set of texture features, based on an adaptive weight function that gives extra weight to matrix elements that in a statistical sense discriminate well between the two classes.

### Experimental design

Designing a classifier and properly evaluating its performance requires a training set and test set containing a sufficient number of cases (Schulerud *et al*, 1998; Nielsen *et al*, 2008). The training set is used to design the classifier, while the test set is used for evaluating its performance. In the training set, the cases’ outcome is known and actively used to design the classifier. We used a prospective sampling method and patients from each group were randomly assigned to either the training set or the independent test set in 1 : 1 ratio.

### Designing and applying the classifier

In the classifier design phase, two adaptive nuclear texture features and their differences were calculated for each case. Each of the three resulting features were evaluated on the training set using linear discriminant analysis in SPSS for Windows statistical package (Version 14.0, SPSS Inc., Chicago, IL, USA). The feature with highest correct classification rate (CCR) was selected. This single feature was then applied to the test set where the outcome was not known.

## Results

Patient characteristics and the results of DNA ploidy analysis are shown in Table 1. Of the 120 patients, 112 were successfully analysed. Gender and race did not differ significantly among groups. There was a significant difference in mean age between group A (55 years (range 28–81)) and group B (70 years (range 47–84)). Barrett's segment length ranged from 1 to 15 cm and was significantly lower in patients in the non-dysplastic group. There was a significant difference in the presence of DNA ploidy abnormalities, with no patients displaying aneuploidy in the non-dysplastic *vs* 65% in the dysplastic group.

### Adaptive features

**Figure 2:**
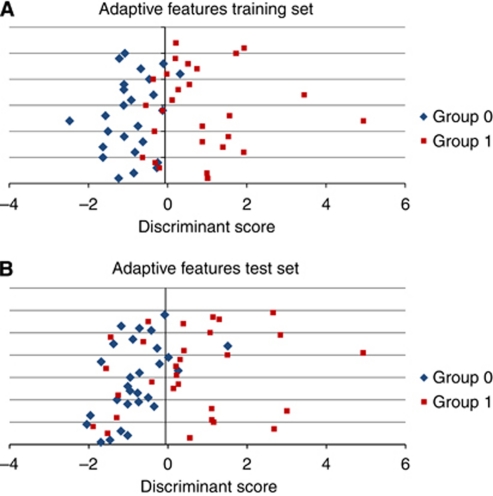
(**A**) Graph demonstrating NT model discirminant analysis on the training set. (**B**) Graph demonstrating NT model discriminant analysis on the validation set.

The selected adaptive feature's CCR was 83% (see Figure 2A). Applying the adaptive feature to the reserved blinded validation set, gave a CCR of 78%. When all 112 patients were evaluated, the sensitivity and specificity for dysplasia by NT was 71% and 93%, respectively. This compared to a sensitivity of 70% and specificity of 100% for DNA ploidy abnormalities. When analysis was undertaken combining both markers in a panel, the overall sensitivity was 76%, specificity 93% and CCR=84%.

A Pearson *χ*^2^ analysis investigating the relationship between ICM positivity and nuclear texture positivity in relation to dysplasia was performed (see Table 2a and b). There was a significant correlation for both ICM (Pearson *χ*^2^=53.2; *P*<0.001) and NT (Pearson *χ*^2^=46.6; *P*<0.001) with the presence of HGD. The combination score of both ICM and NT (see Table 2c) had the highest correlation with dysplasia (Pearson *χ*^2^=53.5; *P*<0.001).

### *Post hoc* misclassification analysis

As noted above, NT classified some of the non-dysplastic in the dysplastic category and some of the dysplastic in the non-dysplastic category. We set out to investigate these patients further and evaluate whether the apparently false classification had any clinical or histological relevance.

Thirteen patients in the dysplastic group were classified incorrectly. Sampling error may be a contributing factor as we did not micro-dissect out areas of HGD, and if there was a very small focus of dysplasia, the population of abnormal nuclei would be relatively small and may not have been identified. The use of micro-dissection to remove only the epithelium of interest has previously been described but this was necessary due to the use of larger prostate core biopsy samples (Pretorius *et al*, 2009). The use of single sections from routinely collected FFPE biopsies makes this a more practical technique for wider application.

Four cases from the non-dysplastic BO group were misclassified as dysplastic by NT. Two patients were indefinite for dysplasia on biopsies 2 years later and a third patient had aneuploidy but no dysplasia on biopsies 3 years later. The fourth patient had no dysplasia or DNA ploidy abnormality after 3 years follow-up. It therefore remains to be seen whether these patients had sub-microscopic changes that could not be assessed by histopathology.

## Discussion

We describe a method of measuring large-scale genomic instability, using nuclear texture analysis to assess chromatin structure and organisation both quantitatively and qualitatively. Our results show that NT can accurately differentiate patients into low- and high-risk subsets by the presence or absence of HGD. Our method uses digital image analysis on the same nuclei used to assess DNA ploidy, which is advantageous as this can be translated into a single platform biomarker, with potential for automation and high throughput. Furthermore, this combination of features was independently tested on a blinded validation set, therefore, reducing errors of bias or overfitting of data that are inherent to other statistical models.

Current published guidelines for the surveillance of BO recommend a random biopsy sampling method to categorise patients by histological grade. This approach is based on analysis of retrospective population studies on neoplastic progression, and the emergence of endoscopic therapy to treat dysplastic BO at an early and curable stage (Overholt *et al*, 2005; Shaheen *et al*, 2009; Pouw *et al*, 2010). Yet, the presence of dysplasia is an imperfect marker of risk of progression to cancer due to issues of lack of compliance with surveillance, biopsy sampling error and inter-observer variability for dysplasia assessment. Conversely, the absence of dysplasia is an imperfect marker of disease quiescence, as the normal morphological appearance of non-dysplastic BO may harbour multiple genetic alterations, which have been shown to increase cancer risk. This may lead to false reassurance that the patient has low risk of progression and delay effective treatment. Molecular changes may, therefore, represent a better method of risk stratification in BO.

Genomic instability has great potential as a biomarker in BO as it is a common finding in oesophageal adenocarcinoma and increases in prevalence through the metaplasia–dysplasia–carcinoma sequence (Chaves *et al*, 2007). DNA ploidy abnormalities are a measure of chromosomal instability, yet despite evaluation in prospective phase 4 biomarker trials, their use has not been adopted routinely (Reid *et al*, 2000b, 2001; Rabinovitch *et al*, 2001; Galipeau *et al*, 2007). This may be explained by the technical difficulty, inter-laboratory reproducibility and cost of using flow cytometry to analyse DNA content. There may also be reluctance to rely on a single biomarker alone, as a panel of biomarkers may more accurately define an individual's future cancer risk. This has been eloquently described by Reid's group who, using a chromosomal instability panel combining 9p loH, 17p loH and DNA content abnormalities, demonstrated that the combination of all three was a better predictor of oesophageal adenocarcinoma than any one biomarker alone (relative risk=38.7; 95% CI=10.8–138.5; *P*<0.001) (Galipeau *et al*, 2007). This panel required a combination of platforms, including short tandem repeat polymorphisms for LOH, as well as flow cytometry, which would be difficult to perform outside of specialist research centres. New single platform techniques to measure chromosomal instability, such as SnP and gene chip arrays (Paulson *et al*, 2009), are being developed that may provide rapid throughput of FFPE material, but the accuracy and cost implications for surveillance programmes remain unclear.

When we combined DNA ploidy and NT, we found our CCR was slightly better than either test in isolation. Nucleotyping yields extra information over DNA content, as changes in chromatin organisation may occur in apparently diploid cells. When using the combination score of both tests, there remained some outliers who were misclassified. Some of these may be explained by sampling error as the median length of BO was significantly higher in these patients. Other studies have also suggested that individual clonal size within a Barrett's segment, rather than segment length itself, provides additional prognostic information (Maley *et al*, 2006). As current practice dictates random four-quadrant biopsy, it is difficult to overcome sampling error, although promising new cytological techniques, that sample a larger field of the Barrett's segment are being investigated (Lao-Sirieix *et al*, 2009).

The statistical analysis used to generate each model was complex, using higher-order statistics. When analysing several feature combinations from multiple data points, care must be taken not to introduce errors in statistical analysis by overfitting of data. This can occur when a feature set or parameter values may accurately describe the samples in the training set rather than general properties of the group. Using the separate training and testing sets approach is therefore a necessity, as if the selection procedure in the training phase results in overfitting, this will be demonstrated in the test set. Encouragingly, when using adaptive features (and hence strongly reducing the probability of overfitting), the CCR was similar in both the training and test sets, indicating that our classifier model was not subject to statistical bias by overfitting. In addition, we used the GLEM adaptive features method for the first time in a clinical study. This method was previously shown to outperform the classical static features in a study on the most difficult set of Brodatz texture pairs (Nielsen *et al*, 2004). This unified statistical approach may allow for generalisable interpretation of data in future nuclear textural analysis studies.

The samples chosen for these analyses were diagnosed as either non-dysplastic or HGD. As this was a discovery study testing several textural features on small group of patients, we felt that two histologically distinct groups were necessary for algorithm generation. In order to avoid equivocal diagnoses, we did not attempt to evaluate low-grade dysplasia (LGD), as this is associated with high inter-observer variability between pathologists (Kerkhof *et al*, 2007). Low-grade dysplasia is a heterogeneous group (dysplastic and reactive) and therefore a lot of patient follow-up is required to determine which are true high-risk LGD that progress to HGD/cancer. We also do not have access to a sufficiently sized cohort of patients to carry out our analysis. Low-grade dysplasia is the group that causes the most diagnostic difficulty however, and in order for this methodology to be useful in clinical practice further evaluation of this group would be valuable. Another group that was not evaluated was intramucosal cancer as this group requires EMR specimens to assess the vertical depth of tumour invasion and the presence of lateral or deep margin involvement by carcinoma, which cannot be assessed using standard mucosal biopsies (Lauwers *et al*, 2009).

The type of HGD (i.e. intestinal-type *vs* gastric foveolar-type) may have had a negative effect on the sensitivity of our test. The diagnostic criteria for Barrett's foveolar-type dysplasia have been published since this study was commenced (Mahajan *et al*, 2010). In variance to intestinal-type dysplasia, foveolar-type dysplasia is typified by non-stratified and basally oriented nuclei that have predominately uniform and smooth nuclear contours. Given this, some of the changes one typically uses to grade dysplasia within intestinal-type dysplasia are not applicable. Other factors that may have contributed to confounding bias include age and length of BO, both significantly increased in the dysplastic group. These limitations can only be overcome in a concerted phase 4 prospective multicentre trial involving patients with non-dysplastic BO at baseline with longitudinal follow-up (Pepe *et al*, 2001). A major advantage of NT and ICM is the routine use of FFPE tissue, which allows analysis on archival material and therefore the potential for longitudinal phase 4 studies on large populations already undergoing surveillance.

In conclusion, we have demonstrated that NT adaptive features may contribute with DNA ploidy for the classification of dysplastic *vs* non-dysplastic BO. The textural features used to differentiate normal from dysplastic tissue were similar to those used in studies of other early cancers. Furthermore, when combining ICM and NT, an 84% CCR was achieved. These data demonstrate that combination of ICM/NT is a promising single platform test, which may aid pathologists in the diagnosis of dysplasia and has potential as a novel biomarker for cancer progression.

## Figures and Tables

**Table 1:** Clinical characteristics of patients

		**Group A (non-dysplastic)**	**Group B (dysplastic)**	***t*-test**
Number of patients analysed		54	58	
Age (years)	Mean (±s.d.)	54.7±11.4	69.6±9.2	*P*<0.001
Gender	Male/female	42/12	49/9	*P*=0.42
Barrett's length (cm)	Mean (±s.d.)	4.8±2	7.0±3.8	*P*<0.001
DNA ploidy abnormality	%	0	65	*P*<0.001

**Table 2:** Results of confusion matrices and *χ*^2^ analysis evaluating both methods

	**Nucleotyping – Pearson *χ*^2^=46.6**			**ICM – Pearson *χ*^2^=53.2**			**Combined score – Pearson *χ*^2^=53.5**		
	**NT result**			**ICM result**			**NT and ICM**		
	**0**	**1**	**Total**	**0**	**1**	**Total**	**0**	**1**	**Total**
*Dysplasia*									
No dysplasia	50	4	54	52	0	52	50	4	54
HGD	17	41	58	19	38	57	14	44	58
Total	67	45	112	71	38	109	64	48	112

Abbreviations: HGD=high-grade dysplasia; ICM=image cytometric DNA analysis; NT=nucleotyping.
